# Retrospective comparative clinical study for silk mat application into extraction socket

**DOI:** 10.1186/s40902-019-0199-z

**Published:** 2019-04-12

**Authors:** Ju-Won Kim, You-Young Jo, Jwa-Young Kim, Ji-hyeon Oh, Byoung-Eun Yang, Seong-Gon Kim

**Affiliations:** 1 Department of Oral and Maxillofacial Surgery, Sacred Heart Hospital, Hallym University, Anyang, 14068 Republic of Korea; 2 Sericultural and Apicultural Division, National Institute of Agricultural Science, RDA, Wanju, 55365 Republic of Korea; 3 Department of Oral and Maxillofacial Surgery, Hallym University Kangnam Sacred Heart Hospital, Seoul, 07441 Republic of Korea; 4 Department of Oral and Maxillofacial Surgery, College of Dentistry, Gangneung-Wonju National University, Gangneung, 28644 Republic of Korea

**Keywords:** Guided tissue regeneration, Silk mat, Polytetrafluoroethylene, Probing depth, Bone gain

## Abstract

**Background:**

Silk mats have been approved for clinical trials by the Korean Food and Drug Administration as membranes for guided tissue regeneration (GTR). In this study, silk mat application was compared to high-density polytetrafluoroethylene (dPTFE) membrane application or no membrane group.

**Methods:**

To compare the silk mat group to the dPTFE group or the no membrane group, a retrospective sample collection was conducted. Bony defects were measured at the time of extraction (T0) and then at 3 months (T1) and 6 months after extraction (T2) on a digital panoramic view. Bone gain (BG) was calculated by subtracting from the bony defect at T0 to the bony defect at each follow-up.

**Results:**

The BG at T2 was 2.44 ± 2.49 mm, 4.18 ± 1.80 mm, and 4.24 ± 2.05 mm in the no membrane group, silk mat group, and dPTFE group, respectively. Both membrane groups had significantly higher BG than BG in the no membrane group at T2 (*P* < 0.05).

**Conclusions:**

Both membrane groups showed higher BG than the no membrane group.

## Background

Third molar surgery is the most frequent procedure in the department of oral and maxillofacial surgery [1, 2]. The complications associated with third molar surgery are classified as early-onset and late-onset [3]. Deep pocket formation adjacent to the second molar is a late-onset complication. This periodontal defect is frequent in cases of deeply impacted lower third molar surgery [4, 5]. Although the systemic condition of the patient may influence this complication [6, 7], the local environment in the impacted teeth is the main etiologic factor [5]. This impaired bone defect is associated with a critical-sized bone defect and may require treatment via graft [8, 9].

Collagen-based plugs have been used for ridge preservation and may be used for third molar surgery. Graft with bone substitute is also considered a preventive measure. However, these materials may be a source for postoperative infection and not be helpful. Different types of membranes have been shown to be reliable. Biodegradable or non-degradable membranes have been introduced. Both types of membranes have been shown a similar outcome [10]. The success rate is associated with the presence of membrane exposure [11]. High-density polytetrafluoroethylene (dPTFE) is introduced for cases with a potential risk of membrane exposure [12].

Silk mat is produced from silkworm cocoon by a simple peeling-off method [13, 14]. Silk mat is mainly composed of fibroin and sericin. Because sericin is a hydrophilic bonding protein that is slowly degraded in water, fragmented sericin is released from silk mats continuously [14]. These sericin fragments are helpful for bone regeneration via a cellular-mediated response [14]. Because silk-based materials have been considered bio-inert and cyto-compatible, silk materials are the main source of scaffolds for bone tissue engineering [15]. As fibroin is resistant to biodegradation, silk mat is classified as non-biodegradable and approved for clinical trials by the Korean Food and Drug Administration (KFDA; Approval number: SPENSER-TS101, approved on November 27, 2015).

The aim of this study was to compare the bone regeneration of the silk mat group to the positive control and negative control in the mandibular third molar defect. As a positive control, the dPTFE membrane group was used. Patients who did not receive the membrane were also recruited as negative controls. Accordingly, a comparison between the membrane-applied groups and the no membrane group was performed.

## Patients and methods

### Collection of data

**Fig. 1:**
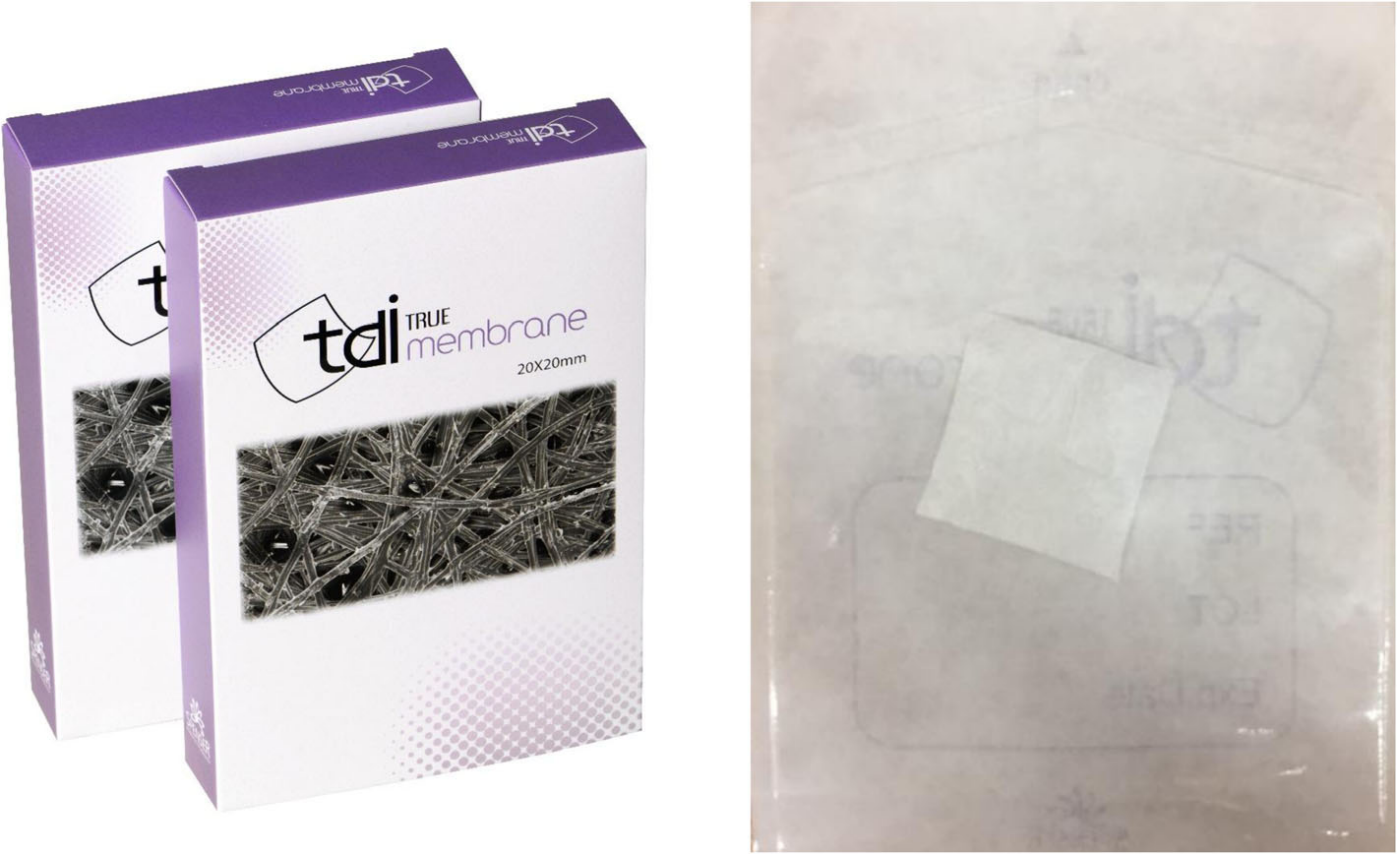
Commercialized silk mat used for this study

Retrospective data collection was performed for comparison with membrane-applied groups. The data for the membrane groups had been collected during previous clinical trials. In previous clinical trials, silk mat was provided by Spencer biomedical technology (Seoul, Korea) (Fig. 1). Cytoplast TXT-200 (Biohorizons, Birmingham, AL, USA) was used for dPTFE membrane. Retrospective unnamed data collection for the no membrane group was approved by the institutional review board of Gangneung-Wonju National University Dental Hospital (IRB No. 2018-003). Clinical trials’ data from the membrane-applied group were provided by Sacred Heart Hospital, Hallym University. This study was performed by chart review. No direct identifiers were recorded on the data sheet. Patients’ chart data were used only for determining inclusion in the analysis. The data in picture archiving and communication systems were also used for evaluating the mandibular third molar and measuring bone defects before and after extraction.

**Fig. 2:**
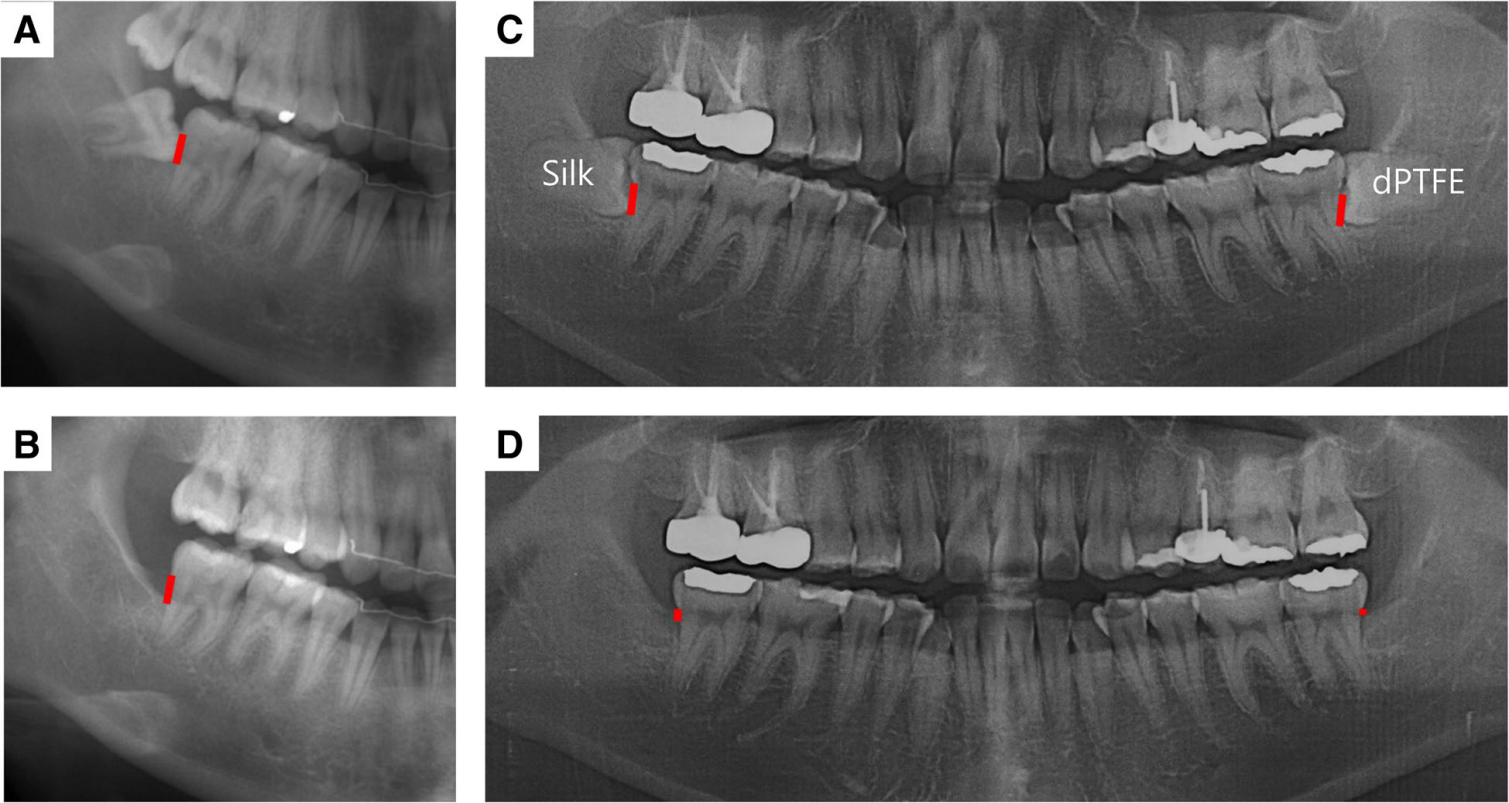
Measurement of the bony defect. Bony defect is the distance between the cement–enamel junction and the bottom of the bony defect on the distal surface of the second molar. The amount of a bony defect is indicated as a red bar on the panoramic view. **a** No membrane group before extraction. **b** No membrane group at 6 months after extraction. **c** Membrane group before extraction. The patient had bilaterally impacted third molar. In this patient, silk mat was applied to the right side and dPTFE to the left side. **d** Membrane group at 6 months after extraction

The inclusion criteria were (1) patients with impacted mandibular third molars, (2) American Society of Anesthesiologists (ASA) physical status I or II, (3) 20 to < 40 years old, (4) present minimum 3-mm bony defect in the distal surface of the mandibular second molar and minimum 5-mm pocket depth in the distal surface of the mandibular second molar at the time of extraction, and (5) patients with a preoperative panoramic view and postoperative panoramic view at either T1 or T2. The exclusion criteria were (1) age < 20 or > 40 years, (2) smokers, (3) patients having any systemic disease, (4) patients receiving irradiation in the head and neck area, (5) patients having malignant cancer history, (6) patients having any oral mucosal disease, and (7) patients having poor oral hygiene. The amount of bony defect was measured on the panoramic view. The distance between the cement–enamel junction and the bottom of the bony defect on the distal surface of the second molar was measured using SigmaScan Pro (SPSS Inc., Chicago, IL, USA) and defined as a bony defect (Fig. 2). Bone gain (BG) was defined as a preoperative bony defect minus a bony defect at follow-up. In cases of T2 samples, any radiogram taken over 12 months after extraction was also excluded.

### Statistical analysis

The comparison of bone level in the same patient at the different observation points of the no membrane group was performed by a paired sample *t* test. An analysis of variance was used in the comparison of BG between the membrane groups and the no membrane group. Bonferroni’s test was used for post hoc analysis. The level of significance was set at *P* < 0.05.

## Results

The numbers of patients in the membrane groups were 25 (average age 24.0 ± 3.6 years, female 18, male 7). All patients from the membrane groups had panoramic views at T0, T1, and T2. For the no membrane group, panoramic views were collected retrospectively. All patients had a panoramic view at T0. The number of patients with panoramic view at T1 was 35 (average age 25.8 ± 4.4 years, female 25, male 10). The size of the bony defect at T0 for this group was 7.35 ± 1.23 mm (Table 1). The number of patients with panoramic view at T2 was 35 (average age 25.1 ± 5.3 years, female 19, male 16). The size of the bony defect at T0 for this group was 7.36 ± 2.12 mm (Table 1). There was no significant difference in patients’ age and sex among the groups (*P* > 0.05). The mean follow-up for the no membrane group at T1 was 3.08 ± 0.37 months, and at T2, it was 7.03 ± 1.44 months. When compared bony defect size in T0 to T1 or T0 to T2 for the no membrane group, the difference between groups was statistically significant (*P* < 0.001).

**Table 1:** Summary of bone defect in patients without graft

Number	T0 (mm)	T1 (mm)	T2 (mm)	*P* value
35	7.35 ± 1.23	5.89 ± 1.26	–	< 0.001
35	7.36 ± 2.12	–	4.92 ± 2.79	< 0.001

*T0* immediate after extraction, *T1* 3 months after extraction, *T2* 6 months after extraction

The size of the bony defect at T0 was 6.47 ± 2.11 and 6.46 ± 2.05 mm in the dPTFE group and the silk mat group, respectively (Table 2). There was no significant difference in the size of the bony defect between groups at T0 (*P* > 0.05). When compared bony defect size in T0 to T1 or T0 to T2 for the membrane groups, the difference between groups was statistically significant (*P* < 0.001).

**Table 2:** Summary of bone defect in patients with membrane

Group	Number	T0 (mm)	T1 (mm)	T2 (mm)	*P* value	
					T0 to T1	T0 to T2
dPTFE	25	6.47 ± 2.11	4.41 ± 1.69	2.23 ± 0.85	< 0.001	< 0.001
Silk mat	25	6.46 ± 2.05	3.89 ± 1.25	2.28 ± 1.13	< 0.001	< 0.001

*T0* immediate after extraction, *T1* 3 months after extraction, *T2* 6 months after extraction

**Fig. 3:**
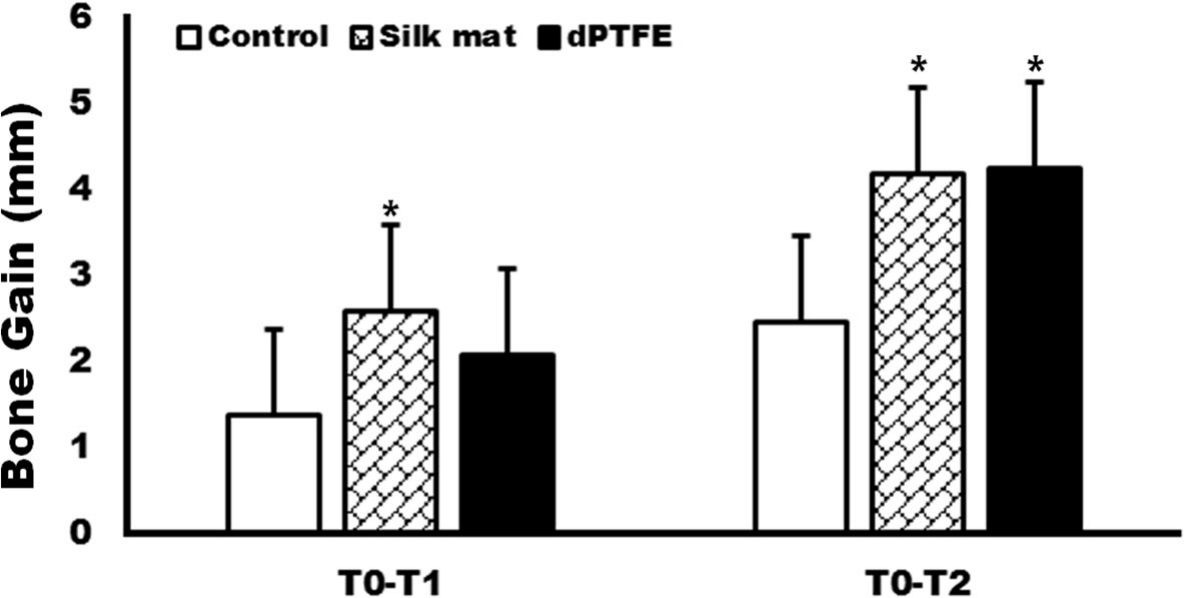
The amount of bone gain (BG) at 3 months (T1) and 6 months after tooth extraction (T2). In a post hoc test, BG in the silk mat group showed a significantly higher gain compared with the control group (nonregenerative/nongraft procedure) at T1. Both membrane groups showed significantly higher BG than the control group (nonregenerative/nongraft procedure) at T2 (*P < 0.05)

The BG in the no membrane group was 1.47 ± 0.50 and 2.44 ± 2.49 mm at T1 and T2, respectively (Fig. 3). The BG in the dPTFE membrane group was 2.06 ± 1.39 and 4.24 ± 2.05 mm at T1 and T2, respectively (Fig. 3). The BG in the silk mat group was 2.57 ± 1.68 and 4.18 ± 1.80 mm at T1 and T2, respectively (Fig. 3). When bone gain was compared among the groups, there was a significant difference at both 3 and 6 months (*P* = 0.008 and 0.002, respectively). In the post hoc test, the silk mat group showed significantly higher BG than the no membrane group at T1 (*P* = 0.006). Both the silk mat group and the dPTFE group showed significantly higher BG than the no membrane group at T2 (*P* = 0.011 and 0.008, respectively). There was no significant difference in BG between the silk mat and dPTFE groups at T1 and T2 (*P* > 0.05).

## Discussion

Silk mat has been widely studied as a membrane for guided tissue regeneration (GTR) [13, 15]. Silk mat shows a similar level of bone regeneration compared to collagen membrane in the animal model [16]. In this clinical study, silk mats showed similar levels of bone regeneration compared to dPTFE membranes (Fig. 3). Both silk mat and dPTFE membrane showed better bone regeneration compared to the non-graft/membrane group (Fig. 3). To the best of our knowledge, this is the first clinical comparative study of silk mats.

The bony defect in the distal surface of the mandibular second molar can be induced by impacted third molar [2]. The size of the defect is dependent on the local environment adjacent to the impacted teeth, such as the degree of impaction, oral hygiene, and systemic disease [2, 4]. If the bony defect after the third molar removal is not reached at the critical-sized defect (CSD), it will be healed without applying graft. To the best of our knowledge, there has been no volumetric analysis for CSD in human third molar surgery. Pocket depth in the distal surface of the second molar has been used as an indicator for CSD determination. In this study, patients with a minimum 3-mm bony defect were included [17]. In our study, the BG for the nonregenerative/nongraft procedure group was 1.4 mm and 2.5 mm at 3 and 6 months, respectively (Fig. 3). The application of PTFE has been shown to improve tissue regeneration compared to the nonregenerative/nongraft procedure group [18, 19]. Additional bone grafts with GTR do not show a synergistic effect after extraction [19].

Silk mat has many benefits compared to other types of membrane. The tensile strength in wet conditions is higher in silk mats than in collagen membranes and dPTFE membranes [16]. Considering the presence of saliva in the oral cavity, the high tensile strength of silk mat in wet conditions is beneficial for clinicians to manage. The production procedure for silk mats is simple [15]. Accordingly, the price for silk mat is also expected to be cheap compared to other types of membrane. The price of material has been an obstacle for the application of membrane in third molar surgery. In addition, silk sericin has bone regeneration ability [20]. Silk mat contains abundant silk sericin as its natural form [14].

In this study, panoramic radiogram was used to measure bone height at the distal surface of the mandibular second molar. Panoramic radiograms have different magnification ratios according to the anatomic site [21]. The shape of jaw bones may also influence image sharpness. Compared to cone-beam computerized tomograms (CBCT), error due to image distortion is higher in panoramic radiograms [21]. However, measurement in panoramic radiographs is highly correlated with that in CBCT and can be used in the posterior alveolus of the mandible [22]. However, CT has three-dimensional and multiple slices of images. Averaging bony defects along the distal surface of the second molar will be time-consuming. As panoramic radiogram is a two-dimensional image, it may have a benefit for measuring the average distance of the overlapped structure. The linear measurements show similar accuracy between CBCT and digital panoramic view [23].

The limitation of this study was that the samples in the control group (nonregenerative/nongraft procedure) were collected separately from 3 months and 6 months because most patients did not have postoperative follow-up radiograms. Accordingly, the controls at 3 months and at 6 months were different. In future studies, the samples for control should also be collected prospectively and in a controlled manner.

## Conclusion

Silk mat showed similar clinical performance to dPTFE when it was used for GTR in the extraction socket. Both membrane groups showed higher BG than the no membrane group experienced.

## Abbreviations

BG: Bone gain
CBCT: Cone-beam computerized tomogram
dPTFE: High-density polytetrafluoroethylene
GTR: Guided tissue regeneration
KFDA: Korean Food and Drug Administration
